# A rapid and affordable point of care test for antibodies against SARS-CoV-2 based on hemagglutination and artificial intelligence interpretation

**DOI:** 10.1038/s41598-021-04298-1

**Published:** 2021-12-30

**Authors:** Vanessa Redecke, Kazuki Tawaratsumida, Erin T. Larragoite, Elizabeth S. C. P. Williams, Vicente Planelles, Adam M. Spivak, Lincoln Hirayama, Marc Elgort, Shane Swenson, Rick Smith, Bryan Worthen, Russ Zimmerman, Patricia Slev, Ben Cahoon, Mark Astill, Hans Häcker

**Affiliations:** 1 Laboratory of Innate Immunity and Signal Transduction, Division of Microbiology and Immunology, Department of Pathology, University of Utah School of Medicine, Salt Lake City, UT USA; 2 Division of Microbiology and Immunology, Department of Pathology, University of Utah School of Medicine, Salt Lake City, UT USA; 3 Division of Infectious Diseases, Department of Medicine, University of Utah School of Medicine, Salt Lake City, UT USA; 4 Associated Regional and University Pathologists (ARUP) Laboratories, Salt Lake City, UT USA; 5 Techcyte Inc., Lindon, UT USA

**Keywords:** Microbiology, Diseases, Health care

## Abstract

Diagnostic tests that detect antibodies (AB) against SARS-CoV-2 for evaluation of seroprevalence and guidance of health care measures are important tools for managing the COVID-19 pandemic. Current tests have certain limitations with regard to turnaround time, costs and availability, particularly in point-of-care (POC) settings. We established a hemagglutination-based AB test that is based on bi-specific proteins which contain a dromedary-derived antibody (nanobody) binding red blood cells (RBD) and a SARS-CoV-2-derived antigen, such as the receptor-binding domain of the Spike protein (Spike-RBD). While the nanobody mediates swift binding to RBC, the antigen moiety directs instantaneous, visually apparent hemagglutination in the presence of SARS-CoV-2-specific AB generated in COVID-19 patients or vaccinated individuals. Method comparison studies with assays cleared by emergency use authorization demonstrate high specificity and sensitivity. To further increase objectivity of test interpretation, we developed an image analysis tool based on digital image acquisition (via a cell phone) and a machine learning algorithm based on defined sample-training and -validation datasets. Preliminary data, including a small clinical study, provides proof of principle for test performance in a POC setting. Together, the data support the interpretation that this AB test format, which we refer to as ‘NanoSpot.ai’, is suitable for POC testing, can be manufactured at very low costs and, based on its generic mode of action, can likely be adapted to a variety of other pathogens.

## Introduction

**Figure 1:**
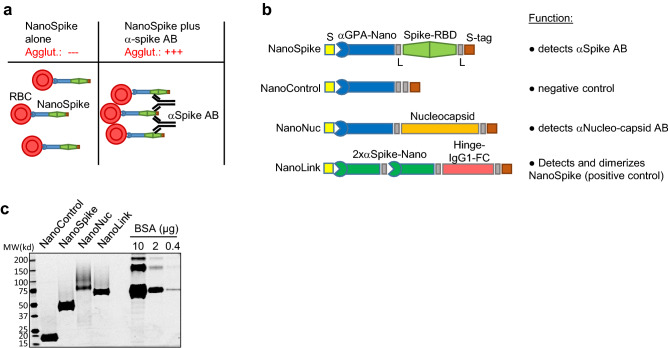
Recombinant proteins and test principle. (**a**) Principle of NanoSpike-mediated hemagglutination in the presence of αSpike AB. (**b**) Schematic presentation of recombinant proteins. S, secretion signal (IFNβ); αGPA-nano, GPA-specific nanobody; L, flexible linker; Spike, Spike-RBD of SARS-CoV-2; S-tag, Tandem-Strep-tag; 2xαSpike-Nano, tandem-fusion construct of two Spike-RBD-binding H11-D4 nanobodies, separated by flexible linker; Hinge-IgG1-FC, dimerizing Hinge-FC moiety of IgG1. (**c**) SyproRuby-stained SDS PAGE of 3 µg of indicated proteins that were expressed in Expi293F cells and purified by one-step affinity purification. Protein yield from 25 ml of Expi293F-cell cultures were 2 mg (NanoControl), 3.75 mg (NanoSpike), 0.55 mg (NanoNuc) and 1.25 mg (NanoLink). Defined amounts of BSA are shown for comparison.

The COVID-19 pandemic caused by SARS-CoV-2 passed its first anniversary with more than 2.5 million deaths worldwide. While initial countermeasures were largely restricted to therapeutic interventions for those with severe infections and prophylactic social distancing, recent clinical studies demonstrated efficacy for various SARS-CoV-2 vaccines, whose deployment is underway in many countries. Still, proper pandemic management hinges on the availability of diagnostic tests, including those revealing the presence of antibodies (AB) against the virus. On a population scale, results obtained by these tests reveal seroprevalence and the dynamics of virus spread, helping to predict susceptibility of a population and guiding health care decisions. On an individual level, such test results indicate previous SARS-CoV-2 infection or successful vaccination and have likely high predictive value for disease susceptibility. While numerous AB tests are available, they do have certain limitations. Tests performed in reference laboratories, such as enzyme-linked immunosorbent assays (ELISA) and related technologies, require special equipment, are relatively expensive and typically associated with long turn-around times from sample acquisition to results. Point-of-care (POC) tests, like the lateral flow immuno-assay (LFIA or LFA), can be conducted with minimal laboratory equipment, but typically show reduced sensitivity compared to tests conducted in reference labs and are also relatively expensive^1–3^. Moreover, analysis is performed by visual inspection, which is subjective in nature and thus affects specificity and sensitivity dependent on more or less conservative interpretation^4^. An alternative format for detection of antigen-specific antibodies in whole blood are hemagglutination tests (HAT). This format is typically based on bi-specific proteins with one moiety, e.g. an AB, binding to red blood cells (RBC) and the other moiety encompassing the antigen, e.g. a virus protein as target for the investigated AB. Antigen-specific AB contained in whole blood bind to the test antigen and mediate visible agglutination of RBC within seconds to minutes (Fig. 1a)^5^. Tests based on such fusion proteins were validated in comparison to a number of FDA-approved assays including ELISA, Western blotting and immunofluorescence assays (IFA), demonstrating remarkably comparable performance^5,6^. A more recent development of this principle employed as RBC-binding moiety a dromedary-derived single variable domain on a heavy chain (VHH)(nanobody) against Glycophorin A (GPA), which is expressed at high levels on RBC^7^. In a proof of principle assay, an E. coli-expressed fusion protein of this nanobody (IH4) and HIV p24 demonstrated high affinity binding of GPA (KD = 33.72 nM) and the detection of p24-directed AB in the plasma of an HIV-positive patient^7^. HAT for AB against SARS-CoV2 have been developed, however, the utility of these tests in POC settings is limited and visual readout remains subjective (see “Discussion” section)^8–10^. Here, we describe the development of a test, which is based on a modified form of the nanobody targeting GPA and the Spike-RBD as antigen. Analyses related to protein production, RBC-binding and hemagglutination in the presence of SARS-CoV-2-specific antibodies suggest excellent test performance. Moreover, artificial intelligence (AI)-based analysis of digital images obtained via cell phone suggest that test interpretation can be objectified and quantified by computer analysis. Given the simplicity, robustness and low production costs, this test may facilitate the assessment of AB responses against SARS-CoV-2 in POC settings, including those with limited resources, and thus contribute to pandemic management.

## Results

### Design, expression and purification of recombinant test proteins

We chose the GPA-directed nanobody IH4 as starting point due to the known robustness of nanobodies and the availability of sequence information^7^. Two mutations were introduced to enable efficient protein secretion in mammalian cells (see “Methods” section). As illustrated in Fig. 1b, we fused the IH4 nanobody initially to two viral antigens, i.e. the receptor-binding domain of the Spike protein (Spike-RBD) of SARS-CoV-2 (NanoSpike) and the Nucleocapsid (NanoNuc). The Spike-RBD was shown to represent a highly immunogenic region in various analyses^11–13^. Antibodies against the Spike-RBD were found to correlate well with neutralizing activity against the virus in both adults and children^14–16^. Moreover, Spike-RBD of SARS-CoV2 did not exhibit cross-reactivity with AB against SARS-CoV or seasonal CoV infections, providing the required predictive value to identify AB against SARS-CoV-2^17^. Last, the majority of currently available vaccines are based on the Spike protein; as such, tests incorporating this protein will allow assessment of successful vaccination. AB against the Nucleocapsid of SARS-CoV2 are also found in the majority of SARS-CoV-2-infected individuals, and AB titers are particularly high in severe cases^14^. Since most vaccines are based on the Spike protein, Nucleocapsid-directed AB can potentially be used to distinguish if AB were obtained during natural infection or vaccination. However, as detailed below, experiments related to test sensitivity (and the potential cross-reactivity of full-length Nucleocapsid with AB against other Coronavirus strains) indicated superior properties of the Spike-RBD as test reagent. As such, we focused our studies primarily on Spike-RBD. In contrast to the original approach using E. coli as expression host, we focused on mammalian protein expression. Spike protein is physiologically targeted to the secretory pathway, which is likely required for proper protein folding and accompanied by glycosylation, which in turn may be relevant for the AB response and test specificity^18^. Thus, to allow for proper targeting to the secretory pathway, we added a secretion signal derived from Interferon-beta to the N-terminus of the fusion protein (Fig. 1b). The Nucleocapsid protein does not contain a secretion signal and is expressed primarily in the cytoplasm. However, initial expression studies suggested increased protein yield and purity when expressed as secreted protein. We therefore followed a similar strategy as used for the NanoSpike and added a secretion signal to the N-terminus (Fig. 1b). A tandem Strep-tag was fused to the C-terminus to allow for efficient one-step protein purification. Flexible glycine-serine-containing linkers were placed between the functional units of the fusion protein to avoid interference of the different domains. A similar protein lacking a viral antigen was cloned as negative control (NanoControl, Fig. 1b). Last, we designed a positive control protein. Here we took advantage of a recently published nanobody against the Spike-RBD, H11-D4^19^. This nanobody was arranged in tandem to increase affinity and linked to the Hinge-FC-domain of IgG1, which forms constitutive dimers. Based on these characteristics, we would expect this protein (NanoLink) to act as surrogate for physiological AB directed against the Spike-RBD and, in the presence of NanoSpike, induce hemagglutination. We expressed these proteins first in small scale, then in larger scale experiments in Expi293F cells (Invitrogen), which are optimized for high-density growth in suspension. Protein expression and Strep-tag-based affinity purification were efficient, resulting in virtually homogenous protein preparations with robust yields (Fig. 1c, Supplementary Fig. 1).

### Functional activity of recombinant proteins

**Figure 2:**
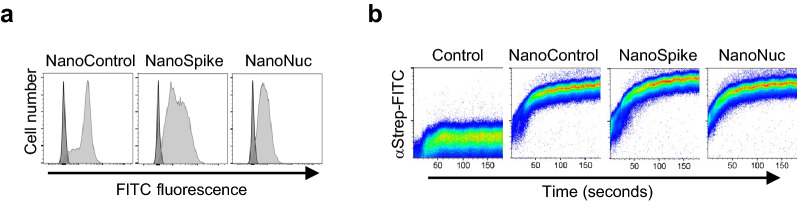
RBC-directed recombinant proteins bind RBC swiftly and quantitatively. (**a**) Flow cytometry analysis of human RBC that were left untreated (dark) or incubated with 30 µg/ml indicated proteins (light), followed by incubation with FITC-labeled AB against the Strep-tag. (**b**) Kinetics of RBC-binding of nanobody-fusion proteins analyzed by flow cytometry. αStrep-FITC AB alone (control) or together with indicated recombinant proteins was added to human RBC (time point 0) and binding was analyzed by flow cytometry during time.

**Figure 3:**
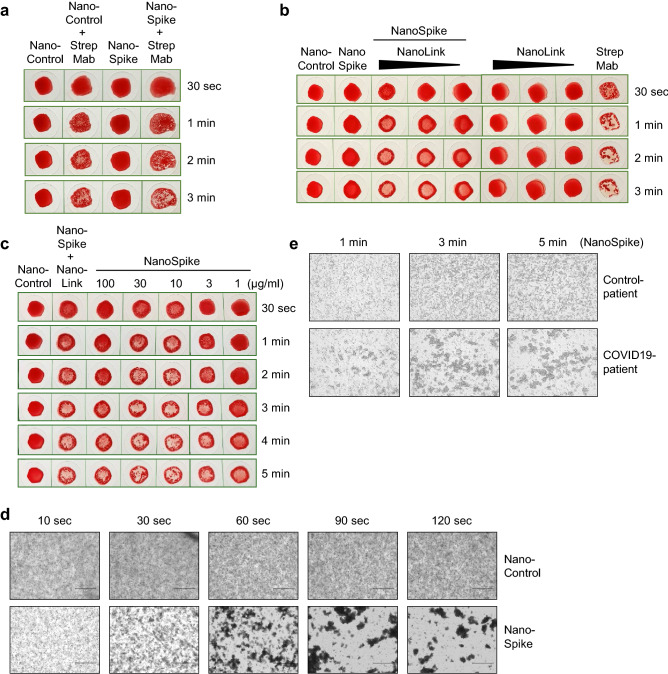
Functional activity of recombinant proteins detecting SARS-CoV-2-specfic AB. (**a**) Hemagglutination assay of whole EDTA blood that was treated with NanoControl or NanoSpike protein (30 µg/ml) in the presence or absence of AB against the Strep-tag. (**b**) Hemagglutination assay of blood that was treated with NanoSpike (30 µg/ml) in the absence or presence of NanoLink at concentrations corresponding to a molar ratio of NanoSpike-to-NanoLink of 9:1, 3:1 and 1:1. Blood treated with NanoLink, NanoControl, NanoSpike or a combination of NanoSpike and αStrep AB is shown for comparison. Time points indicate the duration of agglutination. (**c**) Hemagglutination assay of blood from a convalescent COVID-19 patient that was treated with indicated concentrations of NanoSpike (**c**) or NanoNuc (**d**). Samples treated with NanoControl (30 µg/ml) or NanoSpike (30 µg/ml) plus NanoLink (14.3 µg/ml) serve as controls. Time points indicate the duration of agglutination. (**d**) Phase contrast microscopy of blood from a convalescent COVID-19-patient that was incubated with NanoControl and NanoSpike. (**e**) Phase contrast microscopy of blood from a convalescent COVID-19-patient and a COVID-19-negative control patient, which was incubated with NanoSpike.

We first confirmed binding of recombinant proteins to human RBC by flow cytometry (Fig. 2a). A kinetics analysis illustrates the rapid and quantitative process involved (Fig. 2b). We next tested if AB-mediated cross-linking of NanoSpike triggers hemagglutination. As shown in Fig. 3a, a αStrep-tag AB induced almost instant, clearly visible hemagglutination in the presence of NanoControl or NanoSpike. Neither protein showed any agglutinating activity on its own. Given that NanoControl binds RBC, but lacks a viral antigen, this protein controls efficiently for non-specific (antigen-independent) cross-linking activity. We note that the αStrep AB is a monoclonal IgG1 AB, suggesting that the valency of IgG is sufficient to trigger agglutination.

To simplify the interpretation of the agglutination test, we utilized the positive control protein (NanoLink) as depicted in Fig. 1b. Given its Spike-RBD-binding nanobody (H11-D4) and dimerizing IgG1 FC-domain, this protein is expected to mimic the function of physiological AB directed against the Spike protein and, as a consequence, to induce hemagglutination in the presence of NanoSpike. As shown in Fig. 3b, this is indeed the case. Up to a molar ratio of 9 to 1 of NanoSpike versus NanoLink, NanoLink was inducing clearly visible hemagglutination, while NanoLink alone remained without effect. As such, NanoLink and NanoControl recapitulate the appearance of a negative and positive test results, facilitating visual interpretation.

Having established negative and positive controls, we used blood from a convalescent COVID-19 patient to test if NanoSpike and NanoNuc trigger hemagglutination in the presence of SARS-CoV-2-specific antibodies. For this purpose we titrated the recombinant proteins to establish robust conditions. In a range of 3–30 µg/ml, both, NanoSpike and NanoNuc, triggered hemagglutination, which became visible almost instantly and was fully developed between 1 and 3 min (Fig. 3c,d and Supplementary Fig. 2). Concentrations of recombinant protein above 30 µg/ml reduced agglutination in case of Nanospike, while concentrations below 3 µg/ml were less active in case of both, NanoSpike and NanoNuc. Proteins at 3 µg/ml were active, but induced slightly delayed agglutination in comparison to proteins at 10 and 30 µg/ml (Fig. 3c,d and Supplementary Fig. 2). Neither NanoSpike nor NanoNuc induced hemagglutination at any concentration in the blood of a healthy donor (Supplementary Fig. 3a,b).

Based on these results, we continued our studies with proteins at 30 µg/ml. As expected, macroscopic hemagglutination was reflected by microscopically apparent formation of RBC-clusters induced by NanoSpike in the blood of a convalescent COVID-19-patient (Fig. 3d). NanoControl did not induce any visible cluster formation in the same blood. Likewise, NanoSpike failed to induce RBC-cluster formation in the blood of a COVID-19-negative control patient (Fig. 3e).

Activity of the proteins was maintained for extended periods of time (up to 16 weeks) when stored in a refrigerator at 4 °C (Supplementary Fig. 4). Storage at room temperature (25 °C) and at 37 °C did also not show a loss in activity when assessed with undiluted serum; five-fold dilution of the serum indicated a slight reduction in activity, suggesting that storage at 4 °C (or subzero temperatures in a freezer) will be advantageous if the proteins are stored for extended period of times. We note that optimization of storage buffers is expected to further improve this aspect.

**Figure 4:**
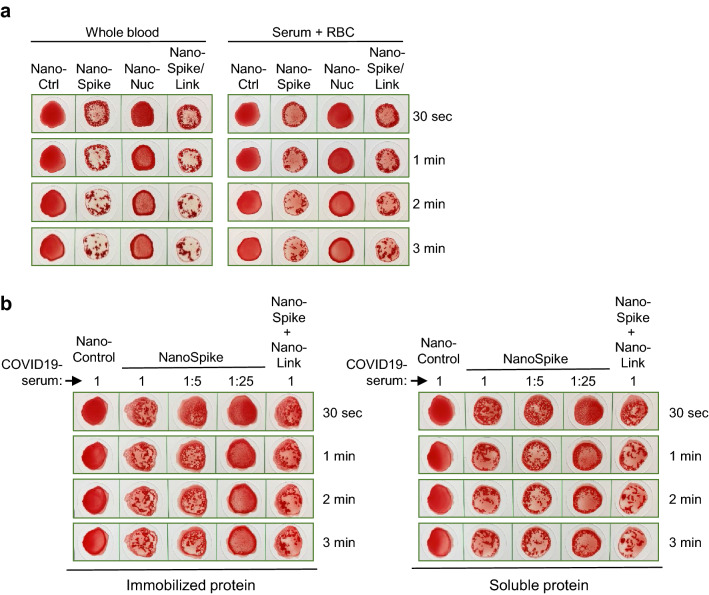
Analysis of whole blood, serum and immobilized proteins in the NanoSpot assay. (**a**) Hemagglutination assay based on samples from a convalescent COVID-19-patient that was treated with indicated proteins. Whole EDTA blood was used directly (left) or serum was isolated and mixed at a physiological ratio with washed RBC from a COVID-19-negative blood group 0 subject (right). (**b**) Hemagglutination assay based on convalescent serum (at indicated dilutions) and recombinant proteins that were either spotted on cardboard (made from 100% extruded polypropylene) and stored at RT overnight (left), or used in their soluble form as described in previous experiments (right). The same cardboard and the same amount of recombinant proteins was used for both conditions.

Given that blood serum can be stored for extended periods of time and is thus more easily available, we tested if serum from a convalescent COVID-19-patient triggered NanoSpike-/NanoNuc-mediated hemagglutination when mixed with washed RBC from a COVID-19-negative healthy subject. As expected, both serum and whole blood containing SARS-CoV-2-specific AB induced very similar hemagglutination (Fig. 4a). Interestingly, spotting and drying of the proteins on the test-cardboard resulted in a hemagglutination reaction that was very similar to application of the proteins in soluble form (Fig. 4b). Given its practical simplicity, this format may be particularly suitable as POC test. Still, experiments employing specialist lab equipment for protein spotting and longitudinal evaluation of protein activity will be required to substantiate this possibility.

Taken together, RBC-targeting nanobody constructs containing SARS-CoV-2-derived antigens bind swiftly and quantitatively to RBC and induce hemagglutination in the presence of virus-specific antibodies. Protein stability is appropriate for a POC environment, and preliminary analyses using cardboard (polypropylene)-immobilized protein suggest feasibility. Given the nature of the proteins used and the visual test appearance, we refer to this test as NanoSpot.

### Analysis of test performance based on sera from COVID-19 convalescent patients and healthy controls

Based on these results, we proceeded to evaluate the test performance of NanoSpot, using a larger number of de-identified samples (serum) from individuals, which had been evaluated at ARUP (Associated Regional and University Pathologists, Inc.) using tests that had received EUA from the FDA for the detection of Spike- and Nucleocapsid-specific antibodies, i.e. an ELISA (EUROIMMUN) and a chemiluminescent immunoassay (CIA)(Abbott), respectively. Hemagglutination was observed for up to three minutes. Using a total of 82 samples, NanoSpike- and ELISA-based tests agreed to 98% for both COVID-19-positive and -negative samples, indicating comparable specificity and sensitivity (Table 1). Using 80 and 108 samples that were CIA-positive and -negative, respectively, NanoNuc showed a negative agreement of 98% and a positive agreement of 74%, indicating high specificity, but reduced sensitivity in comparison to the Nucleocapsid CIA and, likely, NanoSpike. Together, NanoSpike appears to perform comparable to the EUROIMMUN ELISA, while NanoNuc appears overall less sensitive than reference lab tests. Based on these results and the described potential cross-reactivity of the Nucleocapsid protein, we focused on NanoSpike for further development.

**Table 1:** Comparative analysis of NanoSpike- and NanoNuc-mediated hemagglutination tests.

	Nucleocapsid CIA (Abbott)		Spike ELISA (EUROIMMUN)		Positive percent agreement (%)	Negative percent agreement (%)
	Ig pos, n = 80	Ig neg, n = 108	Ig pos, n = 42	Ig neg, n = 40		
NanoSpike			41	39	98	98
NanoNuc	59	106			74	98

Hemagglutination assays based on serum from COVID-19 convalescent and control patients was performed using NanoSpike and NanoNuc and directly compared to results obtained from the Nucleocapsid CIA (Abbot) and Spike ELISA (EUROIMMUN), respectively. Hemagglutination assay controls, i.e. NanoControl and NanoLink were included to aid visual test interpretation.

### Test adaptation to emerging SARS-CoV-2 variants

**Figure 5:**
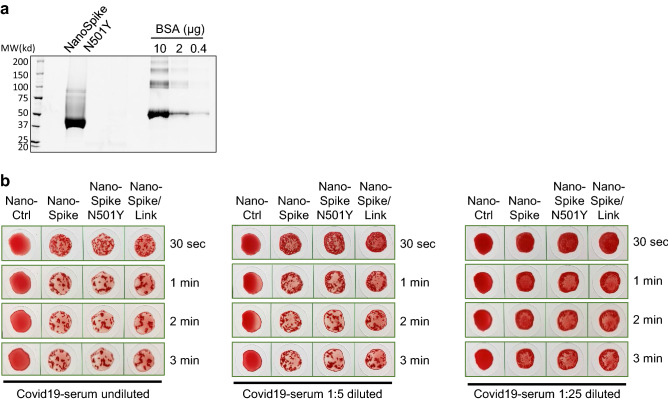
NanoSpot adaptation to emerging SARS-CoV-2 variants. (**a**) SyproRuby-stained SDS PAGE of ~ 20 µg of NanoSpike N501Y, which was expressed in Expi293F cells and purified by one-step affinity purification. Protein yield from 25 ml of Expi293F-cell cultures was 2.5 mg. Defined amounts of BSA are shown for comparison. (**b**) Hemagglutination assay based on samples from a convalescent COVID-19-patient that was treated with indicated proteins. Three dilutions of the serum and three time points were used to reveal potential differences in agglutination activity.

A critical aspect in pandemic management, both from an epidemiological and analytical perspective, is the emergence of novel virus strains with altered characteristics related to transmission, pathogenicity and immunogenicity. Ideally, analytical test formats should entail the flexibility for swift adaptation to such new variants and their changed molecular characteristics. Taking advantage of the simplicity of establishing new protein variants for the NanoSpot assay, we tested this aspect formally by introducing one of the emerging mutations into the Spike-RBD of NanoSpike, i.e. N501Y, which was first identified in a variant of the United Kingdom (B1.1.7). Protein production was comparably efficient to the wildtype (wt) protein (Fig. 5a and Supplementary Fig. 5). Using a wide range of concentrations of convalescent serum, we found that NanoSpike-mediated hemagglutination was comparably efficient between wt and the N501Y mutant protein (Fig. 5b). While this observation is consistent with recent reports demonstrating that human convalescent sera efficiently neutralize viruses with N501Y substitutions, the data also demonstrate that the NanoSpot assay can swiftly be adapted to mutations in the virus genome and possible changes in antigenicity^20–22^.

### Artificial intelligence (AI)-based interpretation for agglutination images

Similar to lateral flow assays, agglutination tests are typically interpreted by visual inspection. Even with the help of the negative and positive controls in the NanoSpot assay, interpretation based on human visual inspection remains subjective and can be affected by the training and expertise of the individual interpreting the test. We thus asked if a more objective method could be developed, and specifically, if an image-based deep-learning algorithm could be trained to identify and interpret hemagglutination on card boards. In its ultimate configuration we envision a scenario where (1) a cell phone app directs the acquisition of the agglutination-test image, (2) the image is immediately uploaded to a server for analysis and interpretation by AI and (3) the AI diagnosis is quickly returned to the cell phone user (see below).

We initially developed a machine-learning algorithm to detect and classify agglutinated and non-agglutinated blood spots in images captured by a cell phone camera (iPhone 11 Pro Max). We trained a deep learning object detection model using 438 positive (agglutinated) and 331 negative (not agglutinated) labels, with an additional 50 positive and 38 negative labels reserved for training validation. This model was configured to identify the location and type (i.e., agglutinated or not) of each blood spot in a captured image along with a confidence score in the interval, [0, 1], for each prediction. Predictions below a predefined arbitrary confidence threshold of 0.2 were discarded as spurious. The resulting model’s performance was then evaluated against a holdout test set composed of an additional 28 positive and 23 negative image captures that had never been shown to the model in training. As shown in Table 2, visual and AI-based test interpretation in the holdout set showed perfect correlation. All visually positive and negative samples were classified correctly by AI. As such, an image-based, AI-driven approach can be used, in principle, to identify hemagglutination on cardboards with high confidence.

**Table 2:** Comparison of visual and AI-based test interpretation.

	NanoSpot (visual readout)		Positive predictive value	Negative predictive value
	pos, n = 28	neg, n = 23		
AI-based test interpretation	28	23	100%	100%

### A semi-quantitative, AI-based approach for evaluation of AB titers

So far, both visual and AI-based approaches have focused largely on qualitative test interpretation, even though visual inspection provides a certain level of relative quantification because test and control samples are directly compared to each other. Such comparative analysis is particularly useful for samples showing minor, spontaneous agglutination, which is observed in rare conditions, such as immune-mediated hemolytic anemia (IMHA)(Supplementary Fig. 6). AI-based interpretation using the algorithm described above will identify such control samples as agglutination-positive with the consequence to invalidate such tests. Given these considerations and the fact that the level of agglutination correlates with AB titers (see below), we hypothesized that AI could be trained to quantify agglutination and, possibly, AB titers. This seemed particularly feasible as samples with negative (or minimal) and positive (or maximum) agglutination are integrated into the test procedure as controls, providing a possible scale for relative quantification.

**Figure 6:**
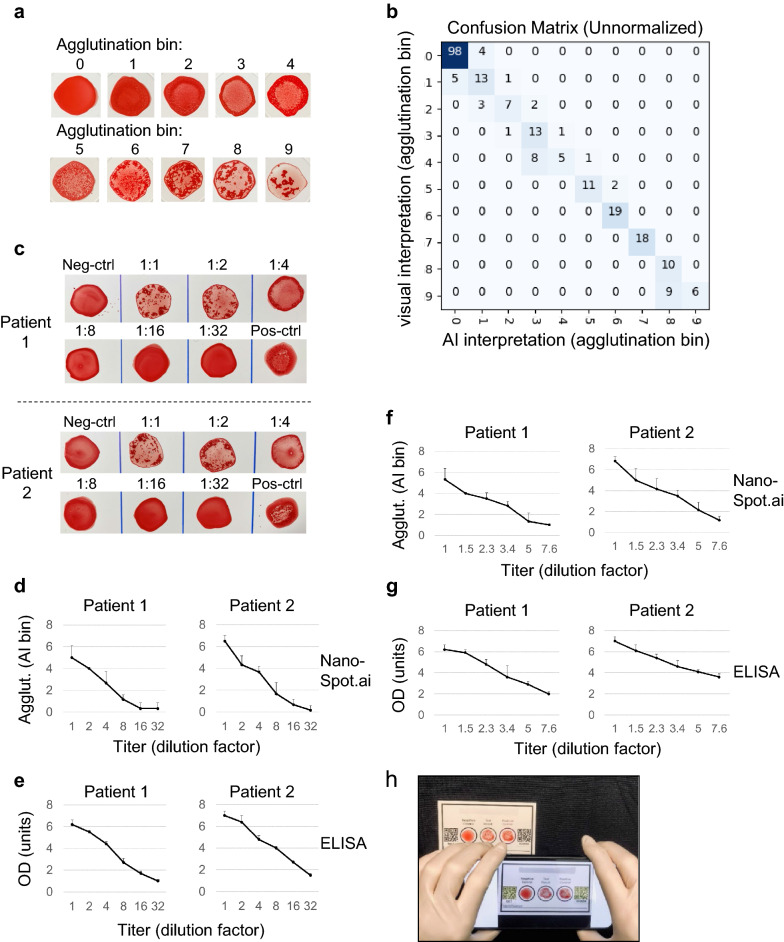
Quantitative assessment of hemagglutination titers using NanoSpot.ai. (**a**) Master palette of hemagglutination samples selected based on visual appearance and reflecting a range of agglutination from 0 (no agglutination) to 9 (strongest agglutination). (**b**) Confusion matrix based on visual and AI-based interpretation of agglutination samples (holdout set), which had not been shown to AI before. (**c**–**g**) Correlation of AB-titer with visual appearance of hemagglutination (**c**), AI-based interpretation (**d**,**f**) and ELISA (**e**,**g**). Serum samples of two COVID-19-positive patients were titrated (in COVID-19-negative serum) and analyzed by indicated methods. Agglutination was terminated when close to fully developed (corresponding to AI-bin 6–8). twofold (**c**–**e**) and 1.5-fold (**f**,**g**) dilution steps were used as indicated. Error bars represent mean ± Std dev based on six (**d**,**f** (AI)) or three (**e**,**g** (ELISA)) replicates. (**h**) Setting used for data acquisition, including test card, cell phone and mobile app capturing image.

In a first attempt towards this quantitative goal, we focused on a step-wise quantification model where a total of 2591 samples were assigned visually to 10 arbitrary bins according to agglutination levels (Fig. 6a), implemented as a generalization from the two original labels, “Agglutination negative” and “Agglutination positive” to 10 new labels: “Agglutination 0” (no agglutination) through “Agglutination 9” (maximum agglutination)(Supplementary Table 1). The samples were then divided into 2326 training and 265 validation labels, followed by training of a deep learning object detection model to identify the location, type (i.e., Agglutination 0–9), and confidence for each blood spot in a manner similar to the previous binary classifier. After training, the model was tested on a holdout set containing 237 samples that had not been used for training. As shown in Fig. 6b, the AI-based method assigned all samples to the same bin or at most one bin apart from those picked by human visual inspection. This is particularly remarkable as agglutination is continuous in nature and assignment of samples to discrete bins by visual inspection was subjective. On the expanded holdout test set, the maximum spurious prediction confidence was 0.41, and the minimum true positive blood spot prediction confidence was 0.53. Thus, for subsequent processing, predictions below a confidence threshold of 0.5 were considered spurious.

In order to characterize directly the relationship between AB titer and level of agglutination (as assigned through AI), we titrated serum from COVID-19 patients, followed by AI-based image interpretation. The EUROIMMUN ELISA was used for comparison. Using two-fold dilution steps, both NanoSpot and ELISA showed a clear, comparable relationship between AB titer and AI-bins (NanoSpot), respectively arbitrary units (ELISA)(Fig. 6c–e). More refined serum titration at 1.5-fold dilution steps showed an approximately linear relationship of AB titer and AI-bins over an eightfold dilution range, with a two-fold dilution corresponding to a distance of ~ 2 AI-bins (Fig. 6f,g). Together, the data suggest that an image acquisition and AI-based interpretation method can be used to identify hemagglutination. Given the relationship between AB titer and level of agglutination, the data also suggest that this method can be used to provide a semi-quantitative assessment of AB titers. Provided the combination of NanoSpot and AI, we refer to this approach as NanoSpot.ai.

### A clinical study for validation of NanoSpot.ai

As noted, our ultimate vision for this test is a scenario where a cell phone app directs the acquisition of the agglutination-test image, that image is uploaded to a server for analysis and interpretation by AI, and the AI diagnosis is returned to the cell phone user. To this end we established testing cards made out of cardboard containing two regions for QR-code stickers for sample identification and camera focusing. We also established an iOS-based mobile app, which initiates a timer upon recognition of the QR-codes (at the start of the agglutination reaction) and captures an image after a selected time frame (to allow for agglutination). An image of the setting, including test card, cell phone and mobile app is shown in Fig. 6h. The captured image is immediately uploaded via the internet to a computer server at Techcyte for analysis. The complete process is shown in Supplementary Movie 1.

Using this pipeline and to validate NanoSpot.ai in a real-world setting, we performed a small clinical study enrolling four groups of patients: (1) patients who had neither been infected by SARS-CoV2 nor had been vaccinated (n = 15), (2) patients who had not been infected, but had been vaccinated (n = 17), (3) patients who had been infected in the past (self-declared positive PCR result), but had not been vaccinated (n = 15) and (4) patients who had been infected (self-declared positive PCR result) and had been vaccinated (n = 10). Patient blood for NanoSpot.ai was obtained by finger-stick. In parallel, venous blood samples were obtained for EUA-approved tests, i.e. a Spike-ELISA (EUROIMMUN), a Spike-CIA (Siemens) and a Nucleocapsid-CIA (Abbot). In addition, to substantiate interpretation of discrepant results, we conducted the so-called S-Flow assay^23^. This flow cytometry-based assay requires a dedicated lab environment and significant effort; however, it detects Spike-specific antibodies with high specificity and sensitivity^23^.

The complete set of the results is provided in Supplementary Table 2. All patient samples were analyzed successfully by all methods with the exception of three samples where image quality obtained by NanoSpot.ai was insufficient or the confidence score was below the set threshold, invalidating the test (NANO_3, _39, _44; Supplementary Fig. 7). We envision that such cases will trigger re-testing once the mobile app is fully developed and that their incidence will be reduced by more extensive AI training and app improvement. NanoSpot.ai tests were ranked positive if analyte and positive controls were two or more bins above negative control, were ranked equivocal if analyte was only one bin above negative control, and were ranked negative if analyte was in same bin or below the negative control.

All group 1 patients (no infection, no vaccination) scored negative in all tests, suggesting comparably high specificity. Likewise, all patients who had been vaccinated (group 2 and 4) were identified as positive by all Spike tests, including NanoSpot.ai. While all group 2 patients (no infection, vaccination) were negative in the Nucleocapsid CIA, some patients of group 4 (infected, vaccinated) were Nucleocapsid-positive (Supplementary Table 2). These results are expected given that the vaccine is based on the Spike protein alone, while natural infection includes both the Spike and Nucleocapsid protein. As such, group 2 and 4 patients are expected to be Spike-positive, while only group 3 and group 4 patients are expected to be Nucleocapsid-positive, dependent on AB titers. The results of group 3 patients (infected, not vaccinated) were more heterogeneous due to variable, in part low AB titers as apparent by low AI-bins (NanoSpot.ai) and arbitrary units (Spike-ELISA, Spike-CIA). The agreement between the different tests is shown in Table 3. Three samples, which were identified as positive by ELISA, NanoSpot.ai and S-Flow assay scored negative by the Nucleocapsid CIA (NANO_23, _45, _57), suggesting false negative results by the CIA (Supplementary Table 2 and Supplementary Fig. S5). All of those samples were close to the detection limit in the ELISA (Supplementary Table 2). As a result, the negative percent agreement (NPA) of the Spike-CIA and both NanoSpot.ai and ELISA was reduced (Table 3). One sample identified as (low) positive in the ELISA (NANO_54) was ranked equivocal in the NanoSpot.ai assay (due to a low positive control), while one sample identified as positive in the NanoSpot.ai assay was ranked negative in the ELISA (NANO_48). The latter sample scored positive in the Nucleocapsid-CIA and S-Flow assay, suggesting correct assessment by NanoSpot.ai (Supplementary Fig. 8). Two ELISA-equivocal samples were also ranked equivocal by NanoSpot.ai, while one ELISA-equivocal sample (NANO_13) was ranked positive by NanoSpot.ai. This sample scored clearly positive in the S-Flow assay, suggesting correct assessment by NanoSpot.ai (Supplementary Fig. 8). As such, while the Spike-CIA appears to be slightly less sensitive in this limited set of patients, ELISA and NanoSpot.ai show very similar performance and overall excellent agreement, particularly when considering the challenging set of low-titer samples (Table 3). This agreement is also apparent in the correlation of signal intensities between the two tests, reflected by a Pearson correlation coefficient of r = 0.83 (Supplementary Fig. 9), further supporting the conclusion that NanoSpot.ai can provide a semi-quantitative assessment of AB titers similar to other test formats. Taken together, this relatively small clinical study indicates that NanoSpot.ai performs with regard to specificity and sensitivity comparably to established EUA-approved AB tests. The data also suggest that a semi-quantitative AB analysis based on the NanoSpot.ai platform is possible.

**Table 3:** Comparative analysis of NanoSpot.ai, Spike-ELISA and Spike-CIA in clinical study.

	Spike ELISA (EUROIMMUN)		Spike CIA (Siemens)		Positive percent agreement (PPA) (%)	Negative percent agreement (NPA) (%)
	Ig pos, n = 35	Ig neg, n = 13	Ig pos, n = 32	Ig neg, n = 16		
NanoSpot.ai	35	12			100	92*
			32	12	100	75*,**
ELISA			32	13	100	81**

*One discrepant sample from a COVID19-positive patient (NANO_48) scored negative by Spike-ELISA and Spike-CIA, but was positive in the Nucleocapsid CIA (Abbot), NanoSpot.ai and S-Flow assay, suggesting that this sample represents a false negative in the Spike-ELISA and Spike-CIA.

**Three Spike-CIA-negative samples (NANO_23, _45, _57) were positive by Spike-ELISA, NanoSpot.ai and S-Flow assay, strongly suggesting false negative interpretation by CIA. One sample (NANO_48) tested negative in the Spike-CIA and Spike-ELISA, but positive by NanoSpot.ai, Nucleocapsid-CIA and S-Flow assay, suggesting false negative interpretation by Spike-CIA and Spike-ELISA.

## Discussion

The data in this paper show that a hemagglutination test based on bispecific proteins can be used to detect SARS-CoV2-specific AB in finger-stick blood. The simple format, inexpensive reagents and fast results suggest particular suitability as POC test. The data further suggest that AI-driven image analysis can be used to identify and quantify hemagglutination on synthetic planar surfaces to determine relative AB titers. While the current App supports uploading and image interpretation, we envision that future versions will return the test result immediately to the cell phone user. The simple modification of test reagents allows quick adaptation to emerging mutant virus strains, providing the flexibility needed to respond to the evolving pandemic.

For the assay described in this paper, two principal modes of test readout are possible, i.e., visual inspection and image/AI-based test analysis. Both take advantage of controls, which provide direct comparatives. While those may be less important for strong agglutination reactions, e.g., upon vaccination, the negative control is critical to differentiate weak positive reactions from subtle, spontaneous agglutination found in some patients. The human eye is highly capable to identify such differences, making correct diagnoses very likely. Still, visual image interpretation is subjective in nature and can thus lead to incoherent test interpretation amongst different investigators. This problem is well recognized in other test formats, such as classic LFAs, which typically rely on visual interpretation^24,25^. To address this issue, we used a deep-learning algorithm to train an AI model on images of agglutination spots obtained by cell phone. In a first attempt, we trained the model only on blood spots with and without apparent agglutination. This attempt was very successful, allowing AI-based identification of hemagglutination with high confidence. However, this plus/minus interpretation did not allow differentiation of mentioned cases of spontaneous agglutination (necessitating invalidation of some tests) and also did not allow for quantification of AB titers. The latter seemed attractive due to the direct correlation of AB-titer and ‘level’ of agglutination. In a proof-of-concept application, we generated 10 bins representing arbitrary levels of increasing agglutination to generate a discontinuous standard curve ranging from non-detectable to maximal agglutination. Training the model on these bins resulted in a remarkable correlation between visual and AI-based image interpretation, ultimately achieving a test performance in a small clinical study that was comparable to standard EUA-approved tests. Importantly, NanoSpot.ai enabled impartial test analysis, free of the subjectivity of individual investigators. Still, we believe that the quantification approach can be improved, in particular through generation of a continuous standard curve (as opposed to the discontinuous bins currently used). This could possibly be accomplished through generation of a regression curve based on existing bin-labeled data or, alternatively, based on de-novo, pairwise comparison of large numbers of spots with different levels of agglutination by human investigators to generate a more fine-grained agglutination ranking scale, followed by re-training of AI. It is likely that such more precise methods will improve overall quantification and, ultimately, allow for more accurate test interpretation, particularly for low-titer cases. As noted, some NanoSpot.ai tests were invalid due to reduced image acquisition quality and consecutive low AI confidence scores. While improvement of the image capture app will likely reduce focusing errors, an important area for improvement will be additional AI training. Specifically, by broadening the spectrum of agglutination samples, including those with imperfections, such as air bubbles or sample preparation issues, AI will be empowered to ignore such artifacts or, if significant, to invalidate the test upon detecting them. Importantly, as opposed to most other tests, the instant notification through NanoSpot.ai and its simple assay format endorses immediate test repetition, thus mitigating undue delays of diagnoses.

Three recent reports used agglutination assays for detection of SARS-CoV-2-specific AB, although in different formats. One focused on antigenic peptides from the Spike proteins, which were chemically linked to a RBC-specific antibody, and agglutination was assessed visually in column agglutination tests (CAT)^8^. Although only few patients were analyzed and sensitivity and specificity, respectively positive and negative agreement with standard tests remain to be evaluated, this format is scalable and can be performed with basic lab equipment. It is, however, neither quantitative nor particularly suitable for POC settings. Two other reports focused on 96 well plate readouts, where agglutination is reflected by morphological changes in blood settling^9,10^. Both reports relied on the Spike-RBD, similar to NanoSpot.ai. One of these reports used RBC-specific single-chain variable fragments (scFv), while the other one used the Glycophorin-A-specific nanobody, similar to NanoSpike. While the former paper was limited to proof-of-principle assays with three COVID-19 patient samples^10^, the latter report involved larger patient numbers, and direct comparison with an approved Spike-RBD CLIA (Siemens) suggested overall comparable test performance^9^. This HAT, which is based on isolated patient serum and washed donor type O RBC, can provide semi-quantitative results through serum dilution. However, this test depends on basic lab equipment, such as pipettes, 96 well plates and donor RBC, and requires a minimum of one hour for test completion. Similar to other visually analyzed tests, test interpretation is subjective. While it seems possible to perform this test with modifications in POC settings based on finger-stick blood, test duration and loss of quantification of the modified format may be limiting factors. Nevertheless, these agglutination-based tests provide interesting alternatives to currently approved AB test formats, particularly in resource-limited settings.

In conclusion, data available so far indicate that NanoSpot.ai could represent a viable alternative to currently used POC tests, combining diagnostic specificity and sensitivity with distinct simplicity, speed, objectivity and, likely, low costs. Not least, given the possibility to exchange the antigen moiety, NanoSpot.ai may serve as a platform to implement further, SARS-CoV2-unrelated AB tests.

## Methods

### Expression vectors

Codon-optimized DNA encoding expressed proteins was synthesized by commercial suppliers (ThermoFisher Scientific/Invitrogen, Synbio, Biomatic) and cloned by standard molecular biology methods into the mammalian expression vector pcDNA3.1(+) (Thermo Fisher Scientific, Invitrogen). All coding sequences were confirmed by Sanger sequencing. To allow for efficient secretion in mammalian cells, the Glycophorin A-specific IH4 nanobody was modified by a F80Y mutation and insertion of a threonine C-terminal of G117 as described previously^26^.

The Spike-RBD (amino acids 328-533) was derived from SARS-CoV-2 isolate Wuhan-Hu-1 (UniProtKB—P0DTC2, SPIKE_SARS2). Full-length Nucleocapsid (AS1-419) was derived from SARS-CoV-2 isolate Wuhan-Hu-1 (UniProtKB—P0DTC9, NCAP_SARS2). The sequence of the Spike-RBD-recognizing nanobody H11-D4 was used as published and fused N-terminal of the human IgG-FC domain as found in Etanercept (16).

### Protein expression

All proteins were expressed in Expi293F cells (ThermoFisher Scientific) using the ExpiFectamine™ 293 Transfection Kit following the manufacturer’s instructions (ThermoFisher Scientific). In brief, cells were cultured at 37 °C and 8% CO_2_ in 25 ml of Expi293 expression medium (ThermoFisher Scientific) in 125 ml bottles on a 25 mm orbital shaker (120 rpm) until reaching a density of 4.5–5.5 × 10e6/ml. Cells were seeded at 3 × 10e6/ml in 25 ml per 125 ml flasks for transfection. 25 µg of DNA and 80 µl of Epifectamine was added to 1.5 ml and 1.4 ml Opti-Plex complexation buffer (ThermoFisher Scientific), respectively. After a 3–5 min incubation at room temperature (RT), the two solutions were combined, gently mixed, incubated for 15 min at RT and added dropwise to cells. After overnight culture, Epifectamine transfection enhancer 1 (150 µl) and Epifectamine transfection enhancer 2 (1.5 ml) was added, and cell suspensions were harvested 2 days later.

### Protein purification

Cell suspensions were centrifuged at 450 g for 5 min and protein-containing supernatant (SN) was cleared using 0.2 µm filter bottles. To remove biotin (interfering with affinity purification), 3 ml of 10 × buffer W (1 M Tris–HCl pH 8.0, 1.5 M NaCl, 10 mM EDTA) was added to SN along with 600 µl of BioLock solution (IBA), followed by ultracentrifugation at 20,000*g* for 20 min at 4 °C. The resulting SN was loaded on columns containing 700 µl of washed Strep-Tactin XT matrix (IBA). The columns were rinsed five times with 1 ml ice-cold PBS, proteins were eluted with 3 × 500 µl of buffer BXT (IBA), concentrated on Amicon Ultra3k concentration columns (3kD cutoff) at 14,000 g at 4 °C and desalted by centrifugation at 1500*g* for 2 min using TBS-equilibrated Zeba Spin desalting columns (0.5 ml, 7 k MWCO, ThermoFisher Scientific). 3–10 µg of purified proteins were analyzed by SDS PAGE and Sypro Ruby staining (ThermoFisher Scientific).

### Hemagglutination

Human de-identified blood and serum samples obtained by venipuncture were used in this study. For whole blood, 10–20 µl of EDTA blood was mixed with an equal volume of the reaction mixture containing recombinant proteins and spread over the test field (~ 1 cm diameter) on agglutination cards. Agglutination cards were from Pro-Lab Diagnostics (Latex Agglutination Cards, used in initial experiments) or YUPO (YUPOBlue, 100% extruded polypropylene, for NanoSpot.ai). For serum, 5–10 µl of serum were added to the same volume of washed RBC of blood group 0. An equivalent volume of the reaction mixture containing recombinant proteins were added and treated as described for whole blood. The mixed sample was swiveled manually and images were taken during time using a cell phone camera. If not stated otherwise, the reaction mixtures contained 30 µg/ml of recombinant NanoSpike, NanoNuc or NanoControl or 30 µg/ml NanoSpike and 14.3 µg/ml of NanoLink.

### Spike IgG ELISA (EUROIMMUN), Nucleocapsid IgG CIA (Abbott) and Spike-RBD IgG CIA (Siemens) for detection of SARS-CoV-2-specific IgG AB

Specimens were tested using the Abbott, Siemens and EUROIMMUN SARS-CoV-2 IgG EUA-cleared immunoassays. The Abbott SARS-CoV-2 IgG assay was performed on the Abbott Architect i2000 (Abbott Laboratories Inc, Abbott Park, IL) according to the manufacturer’s instructions. This is a qualitative chemiluminescent microparticle immunoassay (CMIA) that detects IgG to the Nucleocapsid protein of SARS-CoV-2. According to the manufacturer, there is a direct relationship between the amount of SARS-CoV-2 specific IgG in the sample and the calculated signal of the sample divided by the signal of the calibrator (S/C) index. A result is considered positive if the S/C is greater than or equal to 1.4. The Siemens Healthcare Diagnostics ADVIA Centaur SARS-CoV-2 IgG (COV2G) was performed on the ADVIA Centaur XPT Immunoassay System (Siemens Healthcare Diagnostics Inc. Tarrytown, NY) according to the manufacturer’s instructions. This is a semi-quantitative high-throughput chemiluminescent immunoassay (CLIA) that detects IgG to the S1 RBD region of SARS-CoV-2 Spike. According to the manufacturer, there is a direct relationship between the amount of SARS-CoV-2 antibody present in the patient sample and the amount of relative light units (RLU) detected by the system. A result of reactive or nonreactive is determined according to the Index Value established with the kit calibrators. A sample is considered reactive or positive for S1 RBD IgG when the Index is ≥ 1.00. The EUROIMMUN assay was performed manually according to the manufacturer’s instructions using the Anti-SARS-CoV-2 IgG Enzyme Linked Immunosorbent Assay (EUROIMMUN US, Mountain Lakes, NJ). The assay format is a 96-well microtiter plate coated with SARS-CoV-2 recombinant S1 spike protein produced in HEK 293 cells. Results are calculated as the ratio between the OD of the sample and the OD of the calibrator. This sample to calibrator ratio is interpreted as negative when the ratio is below 0.8, borderline if the ratio is between 0.8 and 1.1, and positive if the ratio is greater than or equal to 1.1. All results were interpreted according to the manufacturer’s cutoffs.

### S-Flow assay

The S-Flow assay was performed as previously described with minor modifications^23^. Briefly, 293 T cells were transiently transfected with an expression vectors for SARS-CoV-2 Spike or control vector. Two days after transfection, cells were harvested and incubated with patient sera diluted 1:625 in FACS buffer (PBS with 0.5% BSA and 2 mM EDTA) for 20 min at 4 °C. After washing, cells were stained with goat anti-human IgG Alexa Fluor 647 (1:200; Thermo Fisher Scientific) and goat anti-human IgM Alexa Fluor 488 (1:200; Thermo Fisher Scientific) for 20 min at 4 °C. Cells were washed, fixed in 4% formaldehyde and analyzed on a BD LSRFortessa flow cytometer for antibody binding. Specific antibody binding was determined based on control-vector transfected cells. Serum was considered test-positive if the percentage of IgG-positive cells of Spike-transfected cells exceeded the percentage of control-vector transfected cells by more than 10%.

### Object detection with deep learning

An object detection network based on YOLOv3 with anchor boxes set to 400 pixels square was initially used to identify agglutinated and non-agglutinated blood spots^27^. Potential training labels were statistically divided into Training and Validation sets in an approximately 9:1 ratio for each class. Training labels were oversampled such that every class had an equal number of presentations to the model in training. The resulting set of training images was presented to the model in each epoch, and the validation images were used to measure training progress after each epoch. Training images were randomly flipped and rotated at 90° intervals, and image hue and saturation were randomly augmented by as much as 10%. The model was trained using stochastic gradient descent with backpropagation using a batch size of 12 and starting with a learning rate of 1e−4 which decreased over 7 epochs to 1e−5. Since training and validation images were selected from a common set of image captures, a holdout test set composed of distinct image captures that were entirely withheld from the model training process was used to evaluate the final trained model for generalized performance. Model predictions consist of object bounding box location and size, object type (i.e., agglutinated blood spot, non-agglutinated blood spot), and confidence value in range, [0, 1]. An arbitrary initial minimum-confidence threshold of 0.2 was used to filter out spurious predictions since the network’s objective function encourages near-zero confidence when no object is present. This initial threshold completely separated valid predictions from spurious object predictions on the test set with the exception of one high-confidence spurious prediction. That prediction was much larger than a valid blood spot in the NanoSpot.ai system and was ignored on that basis.

### Quantitative agglutination assessment using deep learning

For quantitative AI analysis, a total of 2828 agglutination images were assigned visually (by agreement of three investigators) to 10 arbitrary bins according to the level of agglutination (Fig. 6a) ranging from no agglutination at level 0 to maximum observed agglutination at level 9, separated into sets of 2326 training, 265 validation, and 237 holdout labels. Again, the test set was composed of an exclusive set of image captures that were not presented to the model during the training process. The training and validation data sets were used to train a second model using the same architecture and training procedure as the first, with the output being a discrete agglutination level for each spot rather than simply positive or negative identification. When the quantitative model was evaluated on the expanded test set, the maximum false positive confidence was 0.41, and the minimum true positive confidence was 0.53. Thus it was determined that the quantitative model would benefit from a spurious object confidence threshold of 0.5, in contrast to the original arbitrary threshold of 0.2, in order to reduce potential false positive blood spot detections. In addition to filtering spurious predictions by confidence, the standardized layout and geometry of the NanoSpot.ai card enables valid blood spot predictions to be constrained to appropriate sizes and locations, further enhancing the robustness of the analysis process.

### NanoSpot.ai mobile app

We developed an iOS mobile application using the camera and networking capabilities of an iPhone to capture the NanoSpot.ai card images and upload them to the Techcyte AI platform for analysis. Two QR codes, placed on the opposite sides of the card, are used to identify the manufacturing data about the card and provide a unique ID for each test card. The mobile app also uses the QR codes to ensure the image is captured when the orientation and distance to the card are correct and the image is in focus. To ensure that the picture is taken at the correct time, the app scans the QR codes and initiates a countdown timer that notifies the technician when the picture should be taken to produce a valid result. For the clinical study, the mobile app was used in conjunction with an LED illumination box (Photo Studio Box, Depthlan) to maximize the consistency of the test card image captures. The test results were then interpreted on the Techcyte web application. In the future, the results of the test will be sent directly back to the mobile app for presentation at the point of care.

### Clinical studies

Samples were collected under informed consent from healthy donors and individuals with SARS-CoV-2 infection. These studies were approved by the University of Utah Institutional Review Board under the protocols IRB# 131664 and IRB# 00007740, and all methods were performed in accordance with the relevant guidelines and regulations.

## Supplementary Information

Supplementary Legends.

Supplementary Table 1.

Supplementary Table 2.

Supplementary Video 1.

Supplementary Figures.
