# Supplementary material for: A rapid and affordable point of care test for antibodies against SARS-CoV-2 based on hemagglutination and artificial intelligence interpretation
Source: Sci Rep. 2021 Dec 30;11:24507. doi: 10.1038/s41598-021-04298-1 (PMC8718524; doi:10.1038/s41598-021-04298-1)
Supplement: Supplementary file 1 — Supplementary Legends. [file 41598_2021_4298_MOESM1_ESM.pdf]

## Supplementary Table Legends

**Supplementary Table 1 Count of data labels by agglutination level and workflow usage.** Shown are the number of samples for each agglutination level used in training, validation and holdout test.

**Supplementary Table 2 Full set of results from clinical study to evaluate performance of NanoSpot.ai.**

## Supplementary Movie Legends

**Supplementary movie 1 The procedure of NanoSpot.ai.** Test cards with pre-spotted proteins were used.
