# Supplementary material for: A rapid and affordable point of care test for antibodies against SARS-CoV-2 based on hemagglutination and artificial intelligence interpretation
Source: Sci Rep. 2021 Dec 30;11:24507. doi: 10.1038/s41598-021-04298-1 (PMC8718524; doi:10.1038/s41598-021-04298-1)
Supplement: Supplementary file 5 — Supplementary Figures. [file 41598_2021_4298_MOESM5_ESM.pdf]

A rapid and affordable point of care test for antibodies against SARS-CoV-2 based on hemagglutination and Artificial Intelligence interpretation

Vanessa Redecke, Kazuki Tawaratsumida, Erin T. Larragoite, Elizabeth S.C.P. Williams, Vicente Planelles, Adam M. Spivak, Lincoln Hirayama, Marc Elgort, Shane Swenson, Rick Smith, Bryan Worthen, Russ Zimmerman, Patricia Slev, Ben Cahoon, Mark Astill and Hans Häcker

Supplementary Figure 1

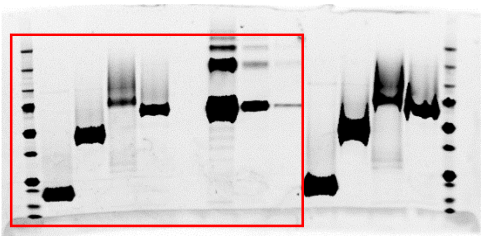

Supplementary Figure 1 Uncropped image of Fig. 1c. Uncropped image of the SDS PAGE shown in Fig. 1c. The red rectangle indicates the relevant area of the image shown in Fig. 1c.

Supplementary Figure 2

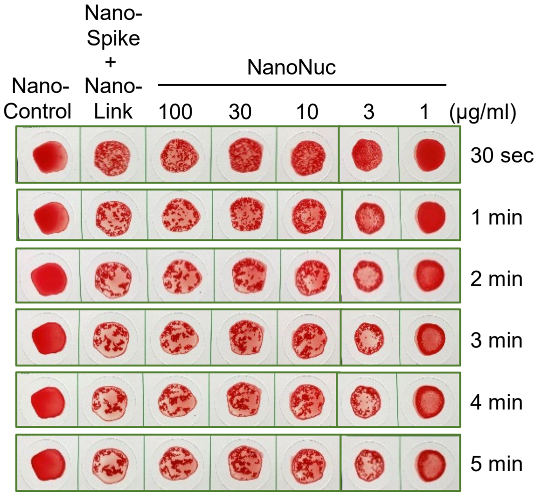

Supplementary Figure 2. Hemagglutination assay of blood from a convalescent COVID-19 patient that was treated with indicated concentrations of NanoNuc. Samples treated with NanoControl (30 µg/ml) or NanoSpike (30 µg/ml) plus NanoLink (14.3 µg/ml) serve as controls. Time points indicate the duration of agglutination.

Supplementary Figure 3

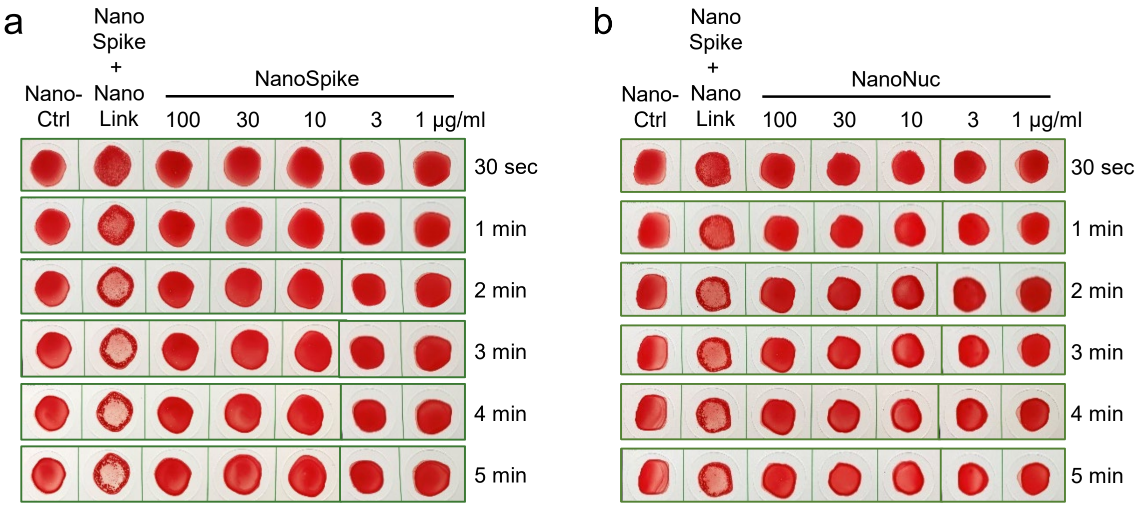

Supplementary Figure 3 Lack of agglutinating function of NanoSpike and NanoNuc in the absence of SARS-CoV-2-specfic AB. (**a,b**) Hemagglutination assay of whole EDTA blood from a COVD19-negative donor that was treated with NanoSpike (a) or NanoNuc (b) at indicated concentrations. Blood treated with NanoControl or a combination of NanoSpike and NanoLink is shown for comparison.

Supplementary Figure 4

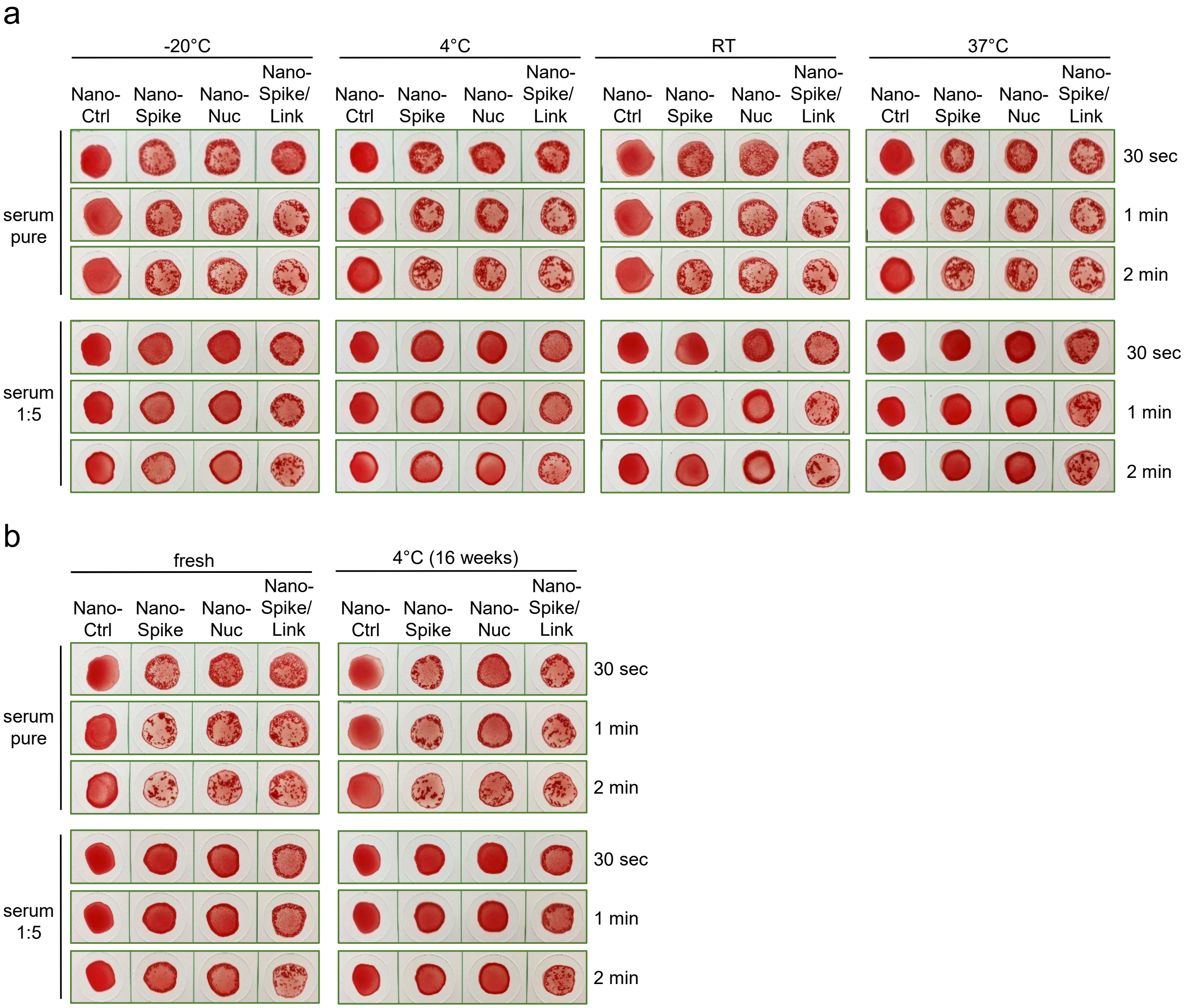

Supplementary Figure 4 Stability of recombinant proteins used in the NanoSpot assay. (**a,b**) Hemagglutination assay based on serum samples (undiluted or 1:5-diluted) from a convalescent COVID-19-patient. The recombinant proteins were stored at the indicated temperatures for four weeks (a) or 16 weeks (b). Proteins stored at -20ºC (a) or freshly prepared proteins (b) served for comparison.

Supplementary Figure 5

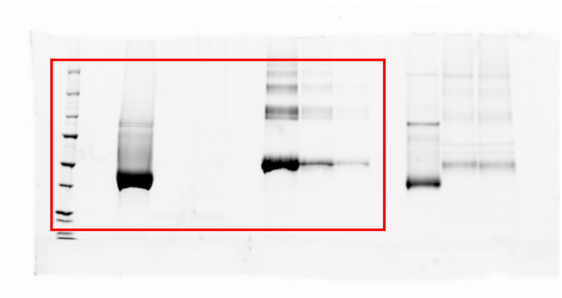

Supplementary Figure 5 Uncropped image of Fig. 5a. Uncropped image of the SDS PAGE shown in Fig. 5a. The red rectangle indicates the relevant area of the image shown in Fig. 5a.

Supplementary Figure 6

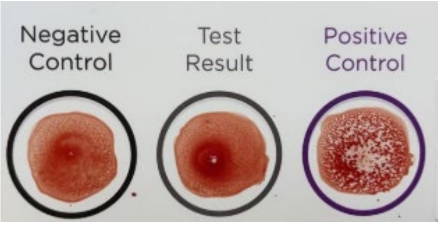

Supplementary Figure 6 Example of low-level spontaneous hemagglutination. Hemagglutination assay of blood from a COVD19-negative donor that was treated with NanoControl (Negative Control), NanoSpike (Test Result) and NanoLink (Positive Control) for one minute. Note the subtle, but clearly visible, agglutination in Negative Control and Test Result samples.

Supplementary Figure 7

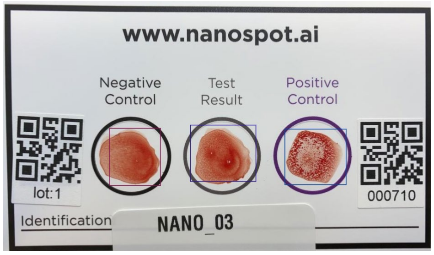

Nano_03
Insufficient focus

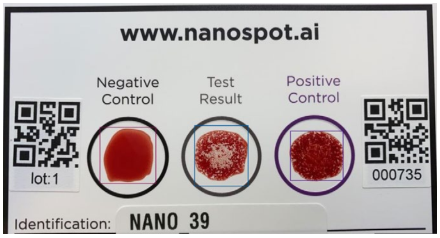

Nano_39
Low confidence score Positive Control (0.29)

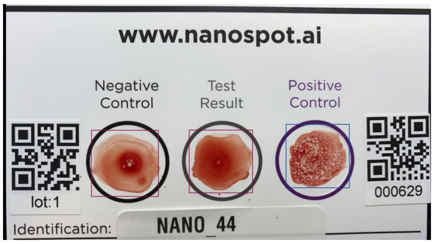

Nano_44
Low confidence score Negative Control (0.36)

Supplementary Figure 7 NanoSpot.ai tests excluded from study due to technical issues

One test with insufficient image quality (blurred focus, Nano_03) and two samples that failed the confidence score of 0.5 (Nano_39, Nano_44) were excluded from test interpretation. Please note that these issues can likely be addressed by improvement of the mobile app (image focusing) and additional training of AI (recognizing artifacts, such as insufficient sample dilution (Nano_39) and air bubbles (Nano_44)).

Supplementary Figure 8

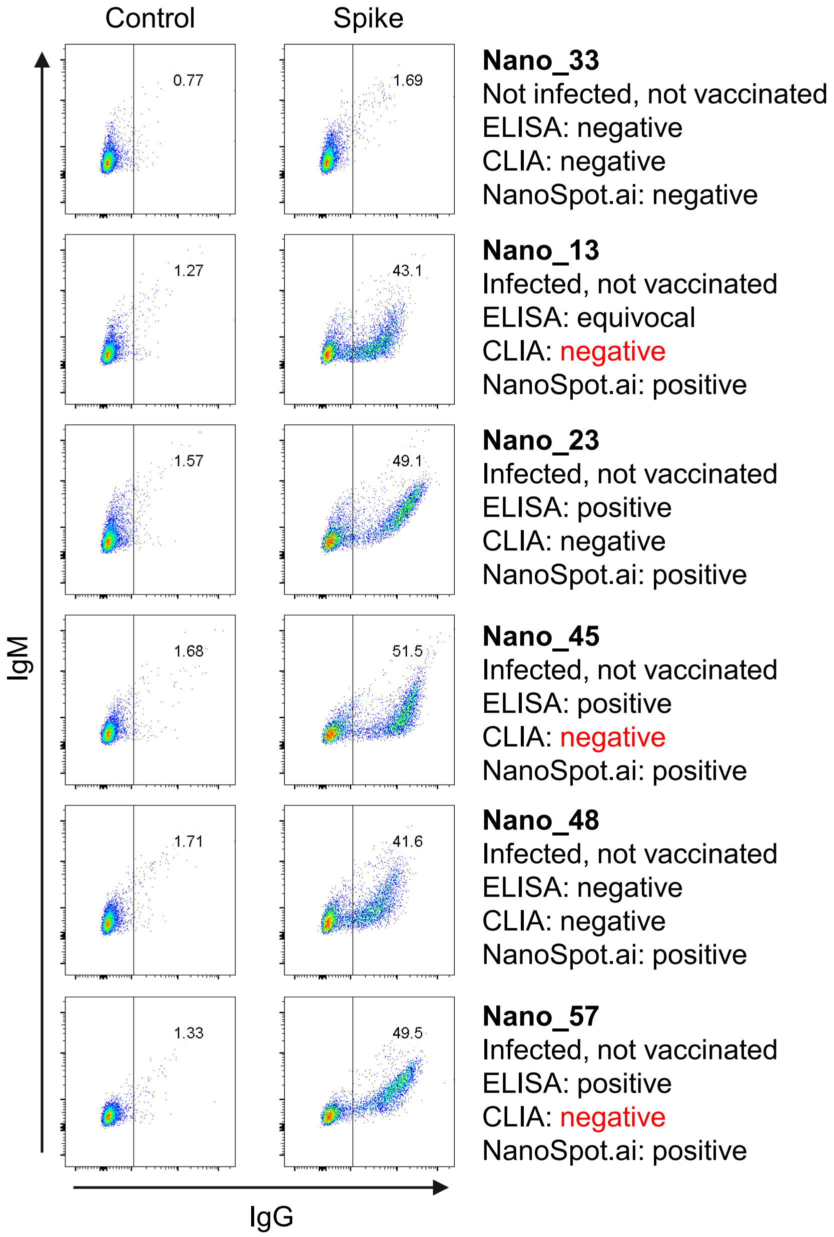

Supplementary Figure 8 S-Flow assay using samples from clinical study

HEK293T cells, which were transfected with a control vector or a SARS-CoV2 Spike expression vector, were incubated with serum from indicated patients, and the presence of αSpike AB was detected by flow cytometry using secondary AB against IgM and IgG, as indicated. Live cells as identified by forward and side scatter are shown.

Supplementary Figure 9

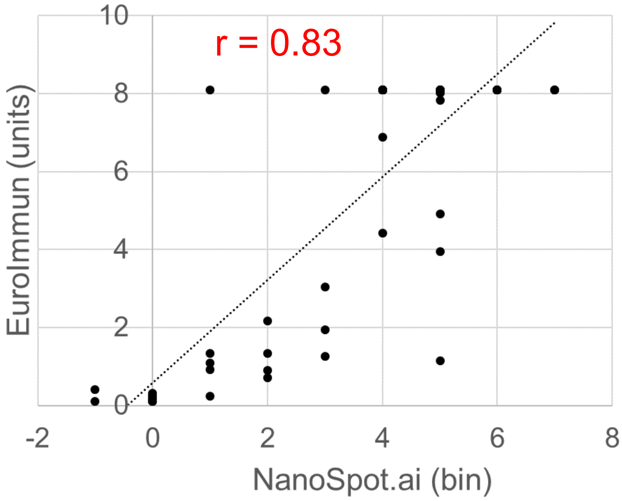

Supplementary Figure 9 Correlation of ELISA and NanoSpot.ai

Results from the clinical study obtained by ELISA (arbitrary units) and NanoSpot.ai (AI bins) were correlated and a trendline was added in Excel. The resulting Pearson correlation index (r), as determined by Excel, is indicated. AI bins obtained by NanoControl were subtracted from AI bins obtained by NanoSpike to eliminate background agglutination.
